# Perioperative circulating tumor DNA in esophageal cancer: prognostic signal in search of a decision framework

**DOI:** 10.1097/JS9.0000000000004672

**Published:** 2026-01-07

**Authors:** Bangbei Wan, Weiying Lu

**Affiliations:** a Reproductive Medical Center, Hainan Women and Children’s Medical Center, Haikou, China; b Department of Urology, Haikou Affiliated Hospital of Central South University Xiangya School of Medicine, Haikou, China

*To the Editor,*

Esophageal cancer carries a high relapse risk despite neoadjuvant therapy (NAT) and esophagectomy. Standard predictors, including radiographic response, ypTNM stage, and tumor regression grade, incompletely reflect residual disease biology^[1,2]^. Circulating tumor DNA (ctDNA) has emerged as a marker of minimal residual disease (MRD) with potential to identify patients at risk for early relapse^[3]^. This commentary was prepared under direct author supervision using language editing support in compliance with the TITAN 2025 guidelines^[4]^.

Shen *et al* present the most comprehensive synthesis of perioperative ctDNA in NAT-treated esophageal cancer to date^[5]^. Across >500 patients, detectable ctDNA after NAT or surgery was associated with significantly worse survival and reduced pathological complete response (pCR), identifying a high-risk subgroup despite multimodal therapy.

## Three findings are most relevant to surgical practice

First, baseline ctDNA was not prognostic. Pretreatment positivity did not correlate with pCR or survival^[5]^. This contrasts with other malignancies and suggests that dynamic clearance rather than a single pre-NAT value better captures treatment sensitivity^[3,6]^.

Second, post-NAT ctDNA defined poor responders. Persistent ctDNA after NAT was associated with approximately 3–4-fold higher risk of progression or death^[5]^. Prospective studies in esophageal squamous cell carcinoma (ESCC) confirm that ctDNA persistence strongly predicts early relapse and may outperform conventional imaging^[7,8]^.

Third, postoperative ctDNA revealed occult residual disease. Detection following R0 resection was associated with a similar increase in relapse risk^[5]^. This mirrors colorectal cancer, where postoperative ctDNA identifies MRD and informs adjuvant therapy strategies^[3,9]^.

Together, these findings suggest a clear model: ctDNA clearance indicates molecular remission; persistence reflects high relapse risk despite favorable pathology.

Methodological strengths include prospective registration, PRISMA reporting, REMARK quality assessment, timepoint-based analysis (baseline/NAT/surgery), and inclusion of modern immunotherapy-based NAT regimens^[5]^. These features increase contemporary relevance.

Limitations merit caution, as all available evidence remains observational with no randomized trials evaluating ctDNA-guided therapy in esophageal cancer. The number of ctDNA-positive cases was modest, introducing statistical uncertainty, and assay platforms varied (digital polymerase chain reaction vs tumor-informed next-generation sequencing) with heterogeneous thresholds for positivity. Moreover, the strongest signals were derived from ESCC cohorts treated with chemoradiotherapy, and it remains uncertain whether similar effects apply to adenocarcinoma in the current era of perioperative chemo-immunotherapy^[1,8]^.

Clinical implications include (1) enhanced risk communication – persistent ctDNA may inform counseling even with favorable imaging; (2) optimized surveillance – positive patients may merit shorter follow-up intervals or early trial referral; and (3) integrated prognosis – combining ypTNM, regression grade, and ctDNA may outperform pathology alone^[3,8]^.

Future priorities include standardization of ctDNA assays, modeling of ctDNA kinetics during NAT, randomized trials of ctDNA-guided adjuvant strategies, multimodal integration with radiomics and pathology, and cost-effectiveness evaluation.

In conclusion, Shen *et al* provide compelling evidence that perioperative ctDNA is a strong prognostic biomarker after NAT and surgery in esophageal cancer^[5]^. While not yet ready for routine use, ctDNA represents a promising tool to shift surgical management toward molecularly informed decision-making.

## Data Availability

No data were used in this Letter to the Editor.

## References

[1] van Hagen P Hulshof MC van Lanschot JJ. Preoperative chemoradiotherapy for esophageal or junctional cancer. N Engl J Med 2012;366:2074–84. 22646630 10.1056/NEJMoa1112088

[2] Shapiro J van Lanschot JJB Hulshof M. Neoadjuvant chemoradiotherapy plus surgery versus surgery alone for oesophageal or junctional cancer (CROSS): long-term results of a randomised controlled trial. Lancet Oncol 2015;16:1090–98. 26254683 10.1016/S1470-2045(15)00040-6

[3] Tie J Wang Y Tomasetti C. Circulating tumor DNA analysis detects minimal residual disease and predicts recurrence in patients with stage II colon cancer. Sci Transl Med 2016;8:346ra392. 10.1126/scitranslmed.aaf6219 PMC5346159 27384348

[4] Agha RA Ginimol M Rasha R. Transparency in the reporting of Artificial Intelligence – the TITAN guideline. Prem J Sci 2025;2. doi: 10.70389/PJS.100082.

[5] Shen T Li T Cao Y Zhang Y Li H. Circulating tumor DNA as a biomarker for progression and survival in esophageal cancer after neoadjuvant therapy and esophagectomy: a systematic review and meta-analysis. Int J Surg 2025;111:8515–22. 40793951 10.1097/JS9.0000000000003017 PMC12626459

[6] Parikh AR Van Seventer EE Siravegna G. Minimal residual disease detection using a plasma-only circulating tumor DNA Assay in patients with colorectal cancer. Clin Cancer Res 2021;27:5586–94. 33926918 10.1158/1078-0432.CCR-21-0410 PMC8530842

[7] Ng HY Jmy K Lam KO. Circulating tumor DNA dynamics as prognostic markers in locally advanced and metastatic esophageal squamous cell carcinoma. JAMA Surg 2023;158:1141–50. 37728901 10.1001/jamasurg.2023.4395 PMC10512170

[8] Hofste LSM Geerlings MJ von Rhein D. Circulating tumor DNA-based disease monitoring of patients with locally advanced esophageal cancer. Cancers (Basel) 2022;14:4417. 36139577 10.3390/cancers14184417 PMC9497103

[9] Tie J Cohen JD Lahouel K. Circulating tumor DNA analysis guiding adjuvant therapy in stage II colon cancer. N Engl J Med 2022;386:2261–72. 35657320 10.1056/NEJMoa2200075 PMC9701133

